# HIV-1 replicates in human osteoclasts and enhances their differentiation *in vitro*

**DOI:** 10.1186/s12977-015-0139-7

**Published:** 2015-02-07

**Authors:** Jin Gohda, Yijing Ma, Ying Huang, Yu Zhang, Lijun Gu, Yang Han, Taisheng Li, Bin Gao, George Fu Gao, Jun-ichiro Inoue, Aikichi Iwamoto, Takaomi Ishida

**Affiliations:** China-Japan Joint Laboratory of Molecular Immunology & Microbiology, Institute of Microbiology, Chinese Academy of Sciences, Beijing, P.R.China; Research Center for Asian Infectious Diseases, The Institute of Medical Science, The University of Tokyo, Tokyo, Japan; Department of Infectious Diseases, Peking Union Medical College Hospital, Chinese Academy of Medical Sciences, Beijing, P.R.China; CAS Key Laboratory of Pathogenic Microbiology and Immunology, Institute of Microbiology, Chinese Academy of Sciences, Beijing, P.R.China; Division of Cellular and Molecular Biology, The Institute of Medical Science, The University of Tokyo, Tokyo, Japan; Division of Infectious Diseases, Advanced Clinical Research Center, The Institute of Medical Science, The University of Tokyo, Tokyo, Japan

**Keywords:** HIV-1, Iinfection, Bone disease, Osteoclasts, Macrophages

## Abstract

**Background:**

HIV-1 infected patients frequently have osteolytic bone disease, which is caused by the dysregulation of the bone remodeling system that involves the interaction between osteoblasts and osteoclasts, but the relationship between osteolytic disease and HIV-1 infection remains unclear. In this study we tested whether HIV-1 infection of osteoclasts affects their differentiation.

**Results:**

We prepared human osteoclasts from CD14^+^ monocytes and examined them for their susceptibility to HIV-1. Furthermore, we investigated the effect of HIV-1 infection on osteoclast differentiation. CD14-derived osteoclasts were shown to express CD4, CCR5, and CXCR4 each at the similar level to that shown with macrophages. R5-tropic HIV-1 and X4-tropic HIV-1 were found to infect CD14-derived osteoclasts and replicate in them. Furthermore, HIV-1 infection induced formation of larger osteoclastst, enhanced the expression of mRNAs for three osteoclast specific marker molecules (tartrate-resistant acid phosphatase, cathepsin K, and the calcitonin receptor), and up-regulated osteoclast bone resorption activity.

**Conclusions:**

Our results suggest that osteoclasts serve as a novel target for HIV-1 infection, which may enhance the osteoclast differentiation contributing to the development of osteolytic disease in HIV-1-infected patients.

## Background

Over the past decades, the use of highly active antiviral therapy (HAART) has succeeded in extending the life span of the HIV-1-infected patients by dramatically reducing mortality among the patients. However, the increased average life span has given rise to long-term complications of HIV-1 infection, resulting in cardiovascular disease, hepatic toxicity, and metabolic disorder [1,2]. Osteolytic bone disease has emerged as one of these complications. Osteolytic bone disease, such as osteoporosis or osteopenia, is characterized by reduction of bone mineral density (BMD). One meta-analysis of prevalence data on osteolytic disease demonstrated that BMD reduction was observed in 67% of HIV-1-infected patients [3]. Although the mechanisms underlying the process of osteolytic disease in HIV-1-infected patients are largely unclear, multiple risk factors are believed to contribute to development of the osteolytic bone disorder. To date, HAART has been shown to cause bone metabolism alterations, leading to BMD reduction [3]. Also, there is clear evidence of reduced BMD in HAART-naïve HIV-1-infected patients [4,5], revealing that HIV-1 infection itself contributes to the development of osteolytic disease.

Bone metabolism is strictly regulated by the bone metabolic turnover, which is referred to as bone remodeling. Bone remodeling consists of the opposing functions of the two cell types, osteoblasts, which produce new bone, and osteoclasts, which resorb old bone. The RANK (Receptor activator of NF-κB)/RANKL (RANK ligand) system dominantly regulates osteoclast formation. RANKL is mainly expressed on the cell surface of osteoblasts and stromal cells, and binding of RANKL to the receptor RANK that exists on the cell surface of osteoclast progenitor cells derived from the monocytic population leads to osteoclast differentiation [6]. Osteoclasts are giant multi-nucleated cells that have the well-developed actin cytoskeleton firmly attached to the bone surface. In the bone of the patients with osteolytic disease, excess formation and abnormal bone resorbing activity of osteoclasts are frequently observed [7]. Interestingly, a recent study shows that HIV-1 transgenic rats undergo severe BMD reduction similar to osteoporosis probably through the enhancement of osteoclast formation [8]. However, it remains unclear how HIV-1 infection alters osteoclast formation and functions, resulting in BMD reduction in HIV-1-infected patients.

In this study we attempted to test the hypothesis that osteoclast differentiation be affected by infection of osteoclasts with HIV-1, since osteoclasts are differentiated from the myeloid-linage progenitor cells, like HIV-1 susceptible monocytes and macrophages.

## Results

### CD14-derived osteoclasts are susceptible to HIV infection *in vitro*

**Figure 1:**
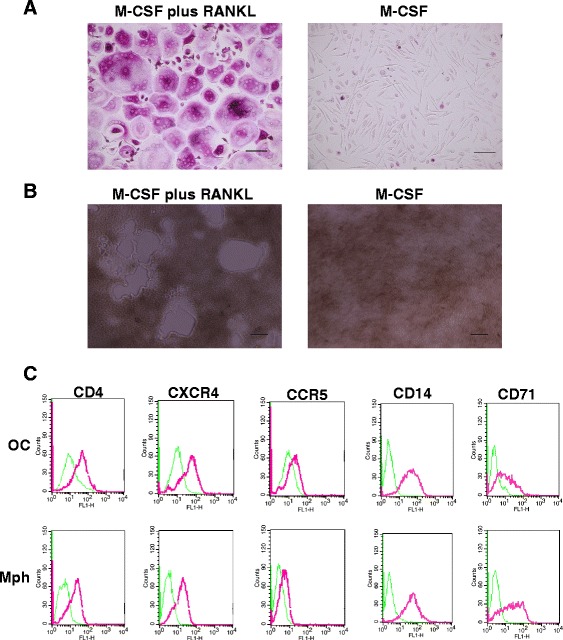
***In vitro*** **osteoclast formation and expressions of the receptors. (A)** CD14^+^ monocytes isolated from the PBMCs from healthy donors were cultured for 7 days with M-CSF plus RANKL, or M-CSF alone to generate osteoclasts and macrophages. The induced osteoclasts (the left photo) and macrophages (the right photo) were fixed and stained for TRAP. TRAP-positive cells appeared as red cells. Scale bar, 100 μm. **(B)** CD14^+^ monocytes isolated from the PBMCs from healthy donors were cultured for 10 days with M-CSF plus RANKL, or M-CSF alone on calcium phosphate-coated wells. After removing the cells, formed pits (white area) were observed by microscopy. Scale bar, 300 μm. **(C)** Osteoclasts (OC) and macrophages (Mph) induced by 7 days culture of CD14^+^ cells with M-CSF plus RANKL or M-CSF alone were harvested and immunostained with anti-CD4, CXCR4, CCR5, CD14, and CD71 antibodies and analyzed by FACS. Pink histograms represent the cells stained with antibodies against each receptor. Green histograms represent the cells stained with each isotype-matched control antibody.

We characterized human osteoclasts generated by culturing with recombinant M-CSF plus RANKL the CD14^+^ monocytes purified from PBMCs [9]. After 6 to 7 days, a large number of osteoclast-like multinucleated cells (MNCs) (Figure 1A). Further incubation with M-CSF plus RANKL induced formation of larger MNCs after 7 to 14 days. The MNCs expressed tartrate-resistant acid phosphatase (TRAP), a specific osteoclast marker [10] (Figure 1A). Furthermore, the MNCs were able to resorb bone in the pit formation assay (Figure 1B). These results indicate that the MNCs were osteoclasts. In contrast, CD14^+^ cells cultured with M-CSF alone expressed a monocyte marker, CD14 at the same level as osteoclasts, and a macrophage specific marker, CD71 at a higher level than osteoclasts (Figure 1C), but not forming any TRAP-positive MNCs (Figure 1A).

We examined expression of CD4, CXCR4, and CCR5, receptors for HIV-1 infection, in CD14-derived osteoclasts and macrophages. Flow cytometry analysis showed that CD14-derived osteoclasts expressed all of the receptors on their cell surface, each at the similar level to that in the macrophages (Figure 1C).

**Figure 2:**
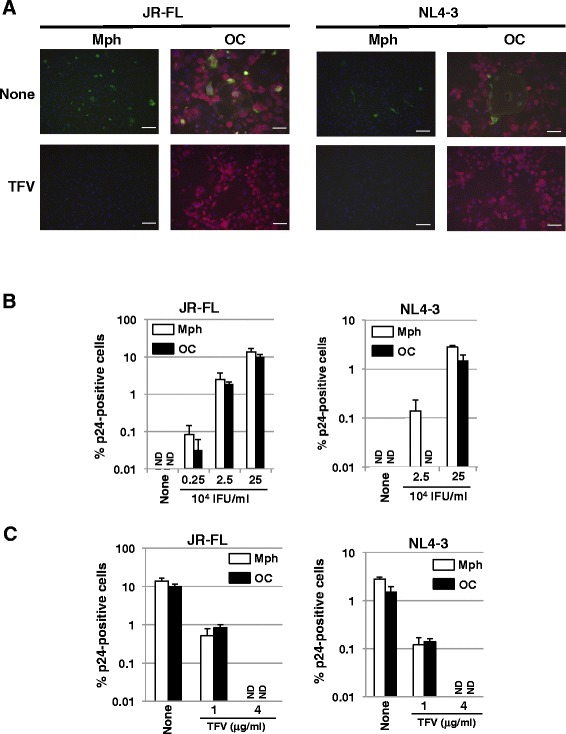
**HIV**-**1 infection of osteoclasts. (A)** The induced osteoclasts (OC) and macrophages (Mph) were incubated for 6 hours with R5-tropic JR-FL (2.5 × 10^5^ IFU/ml) or X4-tropic NL4-3 (2.5 × 10^5^ IFU/ml) in the absence or presence of 4 μg/ml TFV. Forty hours after infection, the cells were fixed and stained with anti-HIV-1 p24 and anti-TRAP antibodies. The green and red signals show expressions of HIV-1 p24 and TRAP, respectively. The blue signals show nucleus stained with Hoechst 33258. Scale bar, 100 μm. **(B)** The induced osteoclasts and macrophages were infected with JR-FL or NL4-3 at the indicated doses. Forty hours after infection, the cells were stained with anti-HIV-1 p24 and anti-TRAP antibodies. The number of p24-positive and negative cells was counted. The bars show the ratio of p24-positive cells to total cells on a logarithmic scale. **(C)** The induced osteoclasts and macrophages were infected with JR-FL (2.5 × 10^5^ IFU/ml) or NL4-3 (2.5 × 10^5^ IFU/ml) in the presence of TFV at the indicated concentrations. Forty hours after infection, the cells were stained with anti-HIV-1 p24 and anti-TRAP antibodies. The number of p24-positive and negative cells was counted. ND means not detected.

We then exposed CD14-derived osteoclasts or macrophages to a CCR5-tropic (R5) HIV-1 strain, JR-FL or a CXCR4-tropic (X4) HIV-1 strain, NL4-3, in order to examine whether or not human osteoclasts are infected by HIV-1. The cells were immunostained with anti-HIV-1 p24 and anti-TRAP antibodies, 40 hours after the infection. In both cases, some macrophages and TRAP^+^-MNCs were found positive with p24 staining (Figure 2A). CD14-derived osteoclasts were infected by JR-FL and NL4-3 as efficiently as the macrophages, though JR-FL infected both CD14-derived macrophages and osteoclasts more efficiently than NL4-3 (Figure 2B). The treatment of the cells with tenofovir (TFV), a reverse-transcriptase inhibitor, prevented JR-FL and NL4-3 infection of CD14-derived macrophages and osteoclasts in a dose-dependent manner (Figure 2A,C).

**Figure 3:**
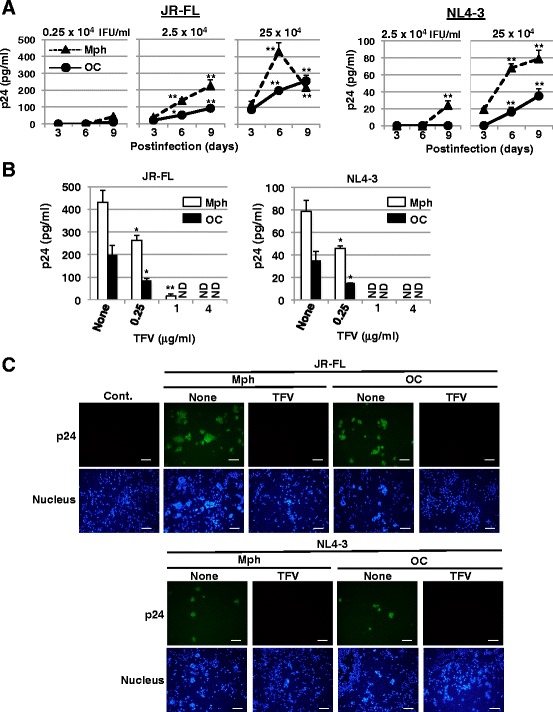
**HIV**-**1 replication in osteoclasts. (A)** The induced osteoclasts (circles) and macrophages (triangles) were incubated for 6 hours with a JR-FL or NL4-3 strain at the indicated doses and washed to remove free virus. Viral p24 concentration in the cell culture supernatants collected on day 3, 6, and 9 was monitored by ELISA. (*p < 0.05, **p < 0.01 by *t* test between day 3 and each day in each cell type) **(B)** The induced osteoclasts (black bars) and macropharges (white bars) were infected with JR-FL (2.5 × 10^5^ IFU/ml) or NL4-3 (2.5 × 10^5^ IFU/ml) in the presence of TFV at the indicated concentrations. Viral p24 concentrations in the cell culture supernatants on day 6 (JR-FL) or day 9 (NL4-3) were measured by ELISA. (*p < 0.05, **p < 0.01 by *t* test between None and each TFV sample) ND means not detected. **(C)** The supernatants from CD14-derived macrophages and osteoclasts infected with JR-FL (2.5 × 10^5^ IFU/ml) or NL4-3 (2.5 × 10^5^ IFU/ml) in the absence or presence of 4 μg/ml TFV were collected on day 9 after infection (shown in Figure 3C). MAGIC5 cells, susceptible to both R5 and X4 HIV-1, were incubated for 6 hours with each supernatant. Three days after infection, the cells were fixed and stained with anti-p24 antibody and Hoechst 33258. The supernatant from the uninfected macrophages was used as a control (Cont.). Scale bar, 100 μm.

Furthermore, the levels of p24 in the supernatants of osteoclast cultures rised in a time-dependent manner, although the p24 levels in CD14-derived osteoclasts were lower then those in macrophages (Figure 3A). The rises of the p24 levels were suppressed by TFV treatment (Figure 3B), and the supernatants had infectivity (Figure 3C), indicating that JR-FL and NL4-3 replicates in CD14-derived macrophages and osteoclasts. Taken together, these data (Figures 2 and 3) indicate that HIV-1 can infect CD14-derived osteoclasts and replicate in them.

### HIV-1 infection enhances osteoclast differentiation

**Figure 4:**
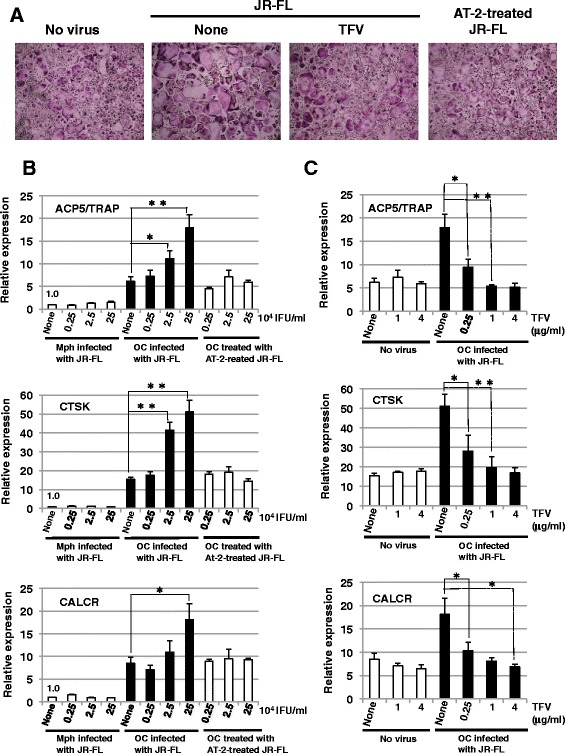
**Effect of R5 HIV**-**1 infection on osteoclast differentiation. (A)** CD14^+^ monocytes were cultured for 6 days with M-CSF plus RANKL. The cells were infected with JR-FL (2.5 × 10^5^ IFU/ml) in the absence or presence of 4 μg/ml TFV or with AT-2-inactivated JR-FL (2.5 × 10^5^ IFU/ml). Five days after infection, the cells were fixed and stained for TRAP. Scale bar, 300 μm. **(B)** CD14^+^ monocytes were cultured for 6 days with M-CSF plus RANKL (OC) or M-CSF alone (Mph). The cells were infected with JR-FL or with AT-2-inactivated JR-FL at the indicated doses. Five days after infection, total RNA was prepared and the expressions of the osteoclast markers, such as ACP5/TRAP, CTSK, and CALCR, were analyzed by quantitative real-time RT-PCR. **(C)** CD14^+^ monocytes were cultured for 6 days with M-CSF plus RANKL (OC) or M-CSF alone (Mph). The osteoclasts were infected with JR-FL in the presence of TFV at the indicated doses. Five days after infection, the expressions of ACP5/TRAP, CTSK, and CALCR were analyzed by quantitative real-time RT-PCR. **(B, C)** The relative mRNA expression level of each marker is shown as fold induction in comparison to the expression in the uninfected macrophages. The data were standardized by the level of β-actin expression in each sample. (*p < 0.05, **p < 0.01 by *t* test).

To elucidate possible link between HIV-1 infection and osteolytic disease, we tested whether HIV-1 infection has any effects on osteoclast differentiation. Microscopic analyses of the TRAP-stained cells revealed that MNCs incubated with JR-FL were significantly increased in size and number of nuclei per cell, in comparison with normal CD14-derived osteoclasts (Figure 4A). In contrast, MNCs treated with TFV or incubated with aldrithiol-2 (AT-2)-inactivated JR-FL were similar to normal CD14-derived osteoclasts.

We further analyzed mRNA expression of specific osteoclast markers, such as acid phosphatase 5 (ACP5) /TRAP, cathepsin K (CTSK), and the calcitonin receptor (CALCR) [11], in the cells infected with different doses of JR-FL or AT-2-inactivated JR-FL (Figure 4B). In the macrophages, these marker expression levels were very low and there was no significant difference in their expressions between the uninfected and infected cells. In contrast, all of the markers were expressed at high levels in the uninfected CD14-derived osteoclasts and further up-regulated by the viral infection in a dose-dependent manner. However, infection with AT-2-inactivated JR-FL did not significantly affect the maker expressions in CD14-derived osteoclasts. TFV treatment suppressed the enhancement of the maker expressions by JR-FL infection in a dose-dependent manner without any effect on the maker expression levels in the uninfected CD14-derived osteoclasts (Figure 4C).

**Figure 5:**
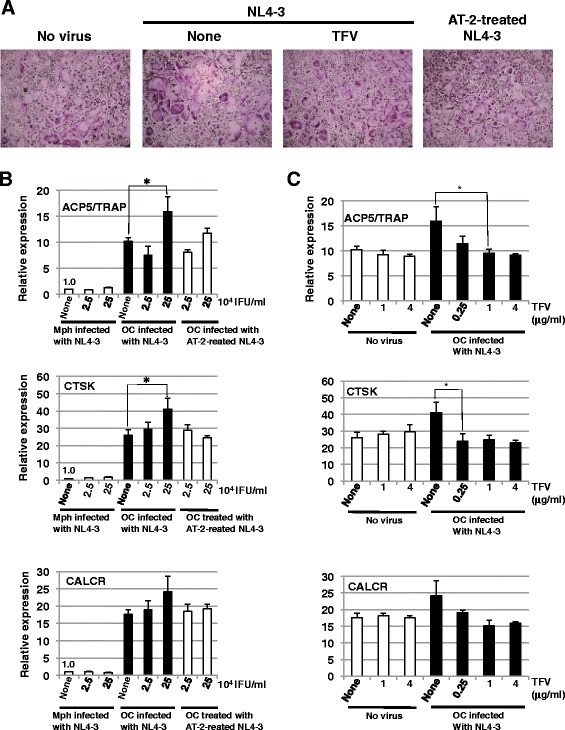
**Effect of X4 HIV**-**1 infection on osteoclast differentiation. (A)** CD14^+^ monocytes were cultured for 6 days with M-CSF plus RANKL. The cells were infected with NL4-3 (2.5 × 10^5^ IFU/ml) in the absence or presence of 4 μg/ml TFV or with AT-2-inactivated NL4-3 (2.5 × 10^5^ IFU/ml). Five days after infection, the cells were fixed and stained for TRAP. Scale bar, 300 μm. **(B)** CD14^+^ monocytes were cultured for 6 days with M-CSF plus RANKL (OC) or M-CSF alone (Mph). The cells were infected with NL4-3 or with AT-2-inactivated NL4-3 at the indicated doses. Five days after infection, total RNA was prepared and the expressions of the osteoclast markers, such as ACP5/TRAP, CTSK, and CALCR, were analyzed by quantitative real-time RT-PCR. **(C)** CD14^+^ monocytes were cultured for 6 days with M-CSF plus RANKL (OC) or M-CSF alone (Mph). The osteoclasts were infected with NL4-3 in the presence of TFV at the indicated doses. Five days after infection, the expressions of ACP5/TRAP, CTSK, and CALCR were analyzed by quantitative real-time RT-PCR. **(B, C)** The relative mRNA expression level of each marker is shown as fold induction in comparison to the expression in the uninfected macrophages. The data were standardized by the level of β-actin expression in each sample. (*p < 0.05 by *t* test).

On the other hand, NL4-3 infection also increased in size and number of nuclei, and enhanced the osteoclast marker expessions, although its effects were less marked as compared with JR-FL infection (Figures 4A,B and 5A,B). Infection with AT-2-inactivated NL4-3 did not affect the cell size and the marker expressions (Figure 5A,B). In addition, TFV treatment inhibited the increase in size and number of nuclei per cell and the enhancement of the marker expression levels (Figure 5A,C).

**Figure 6:**
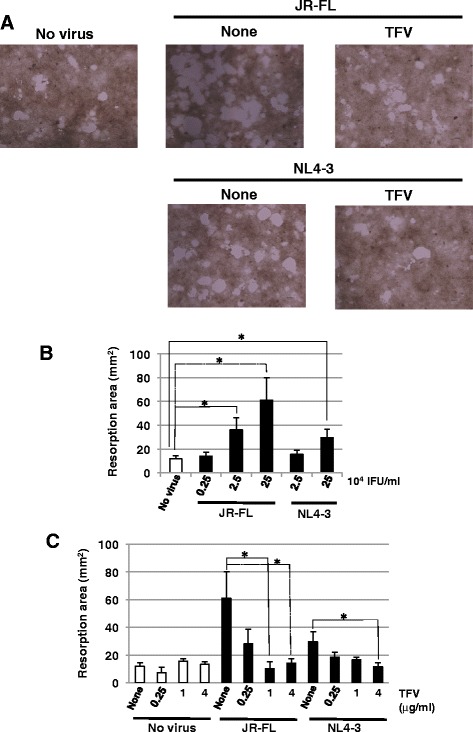
**Effect of HIV**-**1 infection on bone resorption activity of osteoclasts. (A)** CD14^+^ monocytes isolated from the PBMCs from healthy donors were cultured for 6 days with M-CSF plus RANKL, or M-CSF alone on calcium phosphate-coated wells. Then, the cells were infected with JR-FL (2.5 × 10^5^ IFU/ml) or NL4-3 (2.5 × 10^5^ IFU/ml) in the absence or presence of 4 μg/ml TFV. Five days after infection, formed pits (white area) were observed by microscopy. Scale bar, 300 μm. **(B)** The induced osteoclasts (for 6 days culture with RANKL plus M-CSF) were infected with JR-FL or with NL4-3 at the indicated doses. Five days after infection, the pit areas were measured. **(C)** The induced osteoclasts (for 6 day culture with RANKL plus M-CSF) were infected with JR-FL (2.5 x 10^5^ IFU/ml) or with NL4-3 (2.5 × 10^5^ IFU/ml) in the presence of TFV at the indicated concentrations. Five days after infection, the pit areas were measured. (*p < 0.05 by *t* test).

We finally performed a pit formation assay examining the effect of HIV-1 infection on osteoclast bone resorption activity. Microscopic images showed that pit areas produced by JR-FL or NL4-3-infected osteoclasts were larger than those by the uninfected osteoclasts (Figure 6A). Pit formation was enhanced by JR-FL infection and, to a lesser extent, by NL4-3 (Figure 6A,B). Furthermore, TFV treatment suppressed an increase in pit formation by viral infection (Figure 6A,C). Accordingly, these results strongly suggest that HIV-1 infection enhances osteoclast differentiation and bone resoption activity.

## Discussion

We here show that CD14-derived osteoclasts can be also infected with HIV-1 like other myeloid-linage cell types, such as monocytes and macrophages. In addition, HIV-1 can also replicate in CD14-derived osteoclasts. Our data showed that CD14-derived osteoclasts were susceptible to both R5 and X4 tropic HIV-1. R5 HIV-1 (JR-FL) infects CD14-derived osteoclasts and macrophages and replicates more efficiently than X4 HIV-1 (NL4-3). It is unknown why there is a difference in efficiency of infection and replication between JR-FL and NL4-3, although the extent of NL4-3 replication seems to be lower than that of R5 HIV-1 [12]. However, both viruses can infect CD14-derived osteoclasts and macrophages with similar efficiency (Figure 2). On the other hand, HIV-1 less efficiently replicates in CD14-derived osteoclasts compared to macrophages, even though HIV-1 infects both the cell types to the same extent. Why the infection and replication rates are different remains unclear. One possible reason is that osteoclasts might produce a fewer viral particles than macrophages due to the impairment of some step after proviral integration in the HIV-1 life cycle. The further study is needed.

“HIV-1 reservoirs” have recently become a major obstacle in HIV-1 eradication. These reservoirs can escape the antiviral treatment and the host immune response, persisting for a long period of time. Several candidates for HIV-1 reservoirs, including resting CD4+ T cells, monocytes/macrophages, astrocytes, and hematopoietic progenitor cells, have been suggested so far [13]. Although we do not have any evidence that human osteoclasts can be infected with HIV-1 *in vivo*, our results suggest that osteoclasts may act as an HIV-1 reservoir. The limited viral replication in osteoclasts may allow the cells to escape the host immune response.

Osteoclasts are crucial for keeping a normal state of bone homeostasis. Recent studies reported that several markers of bone resorption are markedly increased in HIV-1-infected patients in comparison with healthy individuals [14], suggesting that osteoclasts are involved in the development of osteolytic disorder in the HIV-1 patients. However, osteoclast formation and functions in the bone of HIV-1-infected patients await further investigation. Our data showed that CD14-derived osteoclasts became larger in size with more nuclei in a single cell than normal CD14-derived osteoclasts after incubation with HIV-1. The formation of larger osteoclasts is unlikely to have resulted from syncytium formation induced by HIV-1 infection, because expressions of several osteoclast specific markers, which are closely related to the bone-resorbing activity of osteoclasts [10,15,16], were elevated in CD14-derived osteoclasts incubated with HIV-1. In fact, a pit assay showed that HIV-1 infection enhanced bone resorption activity of CD14-derived osteoclasts. These results strongly suggest that HIV-1 infection enhances osteoclast differentiation and functions. TFV treatment and AT-2-treated inactivation of the HIV-1 replication activity inhibited the enhancement of an increase in cell size and the marker expressions in CD14-derived osteoclasts. In addition, JR-FL infection facilitated osteoclast differentiation more than NL4-3 infection, which is less effective to osteoclasts than JR-FL infection. These indicate that HIV-1 replication might be required for the enhancement of osteoclast differentiation. Taken together, the enhancement of osteoclast differentiation by HIV-1 infection is likely to contribute to BMD reduction observed in HIV-1-infected patients.

Although how HIV-1 infection facilitates osteoclast differentiation remains unclear, direct and indirect mechanism may be involved. A recent study shows that recombinant Tat protein enhances osteoclast differentiation induced by RANKL plus M-CSF, suggesting that Tat protein secreted from the infected cells may affect osteoclast formation [17]. In addition to the viral components, inflammatory cytokines produced by HIV-1 infection may enhance osteoclastogenesis. Formation of osteoclasts and their bone-resorbing activity are enhanced by various kinds of inflammatory cytokines, such as IL-1, IL-6, and TNF-α [18]. Further elucidation of the molecular mechanisms underlying the HIV-1 infection-mediated enhancement of osteoclastogenesis is needed.

## Conclusions

We have shown the susceptibility of CD14-derived osteoclasts to HIV-1 and the enhancement of osteoclast differentiation and function by HIV-1 infection. Thus, these suggest that HIV-1 infection of osteoclasts is one of the causative factors for the development of osteolytic bone disease, such as osteoporosis or osteopenia, in HIV-1-infected patients.

## Methods

### Cell culture and preparation of osteoclasts and macrophages

HEK293FT and MAGIC5 cells were maintained in Dulbecco’s modified Eagle’s medium (DMEM) supplemented with 10% fetal bovine serum (FBS). For preparation of osteoclasts and macrophages, CD14^+^ monocytes were isolated with a purity percentage >95% by using the MACS CD14 Microbeads (Miltenyi Biotech), from peripheral blood mononuclear cells (PBMCs) prepared from two different healthy male donors by Ficoll-Paque (GE Helthcare) density gradient centrifugation. Informed consent for all procedures was obtained from both donors. Cells were cultured for 7 days in Osteoclast Precursor Basal Medium (Lonza) containing 10% FBS with recombinant human macrophage colony-stimulating factor (M-CSF) (33 ng/ml; Peprotech) plus human RANKL (50 ng/ml; Peprotech) or with M-CSF alone. This study was approved by the Ethics Committe of the Institute of Medical Science, The University of Tokyo (Reference number: 24-68-0304).

### Pit formation assay

CD14^+^ monocytes were cultured for 10 days with M-CSF or with M-CSF plus RANKL in Bone Resorption Assay kit 24 (PG Research). After removing cells with 5% NaClO, pit formation was observed with a microscope and photographed. The pit areas were measured with ImageJ [19].

### Tartrate-resistant acid phosphatase (TRAP) staining and immuno-staining of cells and flow cytometry

TRAP staining was carried out with TRAP-staining kit (Primary Cell Co.). For immunostaining, cells were fixed with 4% paraformaldehyde. Then, cells were permeabilized and blocked with PBS containing 2% bovine serum albumin (BSA) and 0.01% Tween 20, and incubated with anti-HIV p24 antibody (clone Kal-1; Dakocytomation) and anti-TRAP antibody (Santa Cruz). After being washed, cells were further incubated with Alexa Fluor 488 anti-mouse IgG (Molecular Probes), Alexa Fluor 546 anti-rabbit IgG (Molecular Probes), and Hoechst33258 (Molecular Probes). The images of fluorescence were acquired by fluorescent microscopy using a BZ-8000 (Keyence). For flow cytometry, cells were harvested with 10 mM EDTA/PBS. After preincubation with a human Fc receptor blocking reagent (MBL), cells were stained with the corresponding antibodies. Fluorescence was analyzed with a FACSCalibur (Becton Dickinson). The following antibodies were used for staining: fluorescein isothiocyanate (FITC) -labeled anti CD4, CD184/CXCR4, CD195/CCR5, CD14, and CD71. In addition, the following isotype controls were used as negative controls for staining for each receptor: FITC-labeled mouse IgG1 for CD4, FITC-labeled rat IgG1 for CXCR4, and FITC-labeled rat IgG2a for CCR5. All of the antibodies except for CD14 and CD71 (Miltenyi Biotech) were obtained from MBL.

### Viral preparations and infection

HEK293FT cells were transfected with a JR-FL [20] or NL4-3 [21] vector using Lipofectamine 2000 (Invitrogen). Two days after transfection, the supernatants were collected and centrifuged to remove the cells. Titration of viral solution was performed by HIV-1 p24 quantification using the HIV-1 p24 ELISA kit (ZeptoMetrix). Infectious units (IFU) of each virus stock were determined by p24 immuno-staining of infected MAGIC5 cells, susceptible to both R5 and X4 tropic HIV-1 [22]. Inactivated viruses were prepared by incubating the viral supernatants with 500 mM aldrithiol-2 (Sigma) at 4°C overnight [23]. For infection of macrophages and osteoclasts, cells were incubated for 6 hours with JR-FL or NL4-3, washed three times with OPBM, and further cultured for the indicated times. Tenofovir (Selleckchem) was added at the indicate concentrations 30 min before infection and added again after washing with OPBM. After detection of infected cells by p24 immuno-staining, the number of infected macrophages and osteoclasts in total 1000 to 3000 cells was counted to determine the infectious rates. The levels of p24 in the supernatants were measured by HIV-1 p24 ELISA (XpressBio). For infection of MAGIC5 cells, cells were incubated for 6 hours with the supernatants in 6 or 9 days cultures of the IR-FL- or NL4-3-infected macrophages and osteoclasts, washed three times with fresh medium, and further cultured for 3 days.

### Quantitative real-time PCR

Total RNA was reverse transcribed with Primescript RT reagent kit (TAKARA) using oligo dT primers. Real-time RT-PCR analysis was performed using a TP870 thermal cycler (TAKARA) and SYBR Premix Ex Taq II (TAKARA). The level of β-actin expression in each sample was used to standardize the data. The following primer sets were used: *ACP5* (5′-GTGTGCAAGACATCAATGACAACAG-3′, 5′-TCTTGAAGTGCAGGCGGTAGAA-3′), *CTSK* (5′-GTCTGAGAATGATGGCTGTGGA-3′, 5′-CATTTAGCTGCCTTGCCTGTTG-3′), *CALCR* (5′-GAACTACGTGACCTCTGCAAGACAA-3′, 5′-AACAGCTAGGTCCTGCCCAATG-3′), and *ACTB*(5′-TGGCACCCAGCACAATGAA-3′, ′-CTAAGTCATAGTCCGCCTAGAAGCA-3′).

### Statistical analysis

The *P* values were calculated with the Student’s *t* test with Microsoft Excel software, with two-tailed distribution and two-sample unequal variance parameters.
